# Routine Chest Computed Tomography at Presentation Does Not Identify Distant Metastasis in cT1aN0 Papillary Thyroid Carcinoma

**DOI:** 10.1089/thy.2020.0073

**Published:** 2020-11-05

**Authors:** Shiori Kawano, Akira Miyauchi, Yasuhiro Ito

**Affiliations:** Department of Surgery, Kuma Hospital, Kobe, Japan.

**Keywords:** papillary thyroid microcarcinoma, active surveillance, chest CT, whole-body search, distant metastasis

## Abstract

***Background:*** Active surveillance is accepted as a first-line management for patients with low-risk papillary thyroid microcarcinoma (PTMC) without metastasis or extrathyroid extension. While the lung is the most frequent site of distant metastasis of papillary thyroid carcinomas (PTCs), it is unclear if chest computed tomography (CT) is necessary at the initiation of active surveillance. In our institution, we usually did not perform chest CT for candidates identified for active surveillance. However, routine preoperative chest CT for patients with thyroid cancers, including PTMCs, was standard practice. The present study aimed to evaluate the clinical value of chest CT for patients with PTMCs.

***Methods:*** We retrospectively reviewed preoperative chest CT images from 1000 consecutive patients (88.5% women; median age, 55 years) with cT1aN0 PTC who underwent surgery between January 2006 and May 2012 in our hospital. The median maximum size of PTMCs was 8 mm.

***Results:*** Abnormal findings were detected in 326 (32.6%) of the 1000 patients. Of these, 290 patients had presumed benign or inflammatory lesions of no clinical importance. In total, 36 patients (3.6%) were referred to specialized departments for further evaluation of the lesions, and 9 patients (0.9%) received invasive tests and/or treatments. Five patients (0.5%) benefited from the chest CT (lung cancer was detected in four, and a cardiac lesion was detected and treated in one), while the lesions in the other four patients were benign, not necessarily requiring treatment. The remaining 27 patents were followed for presumed benign or inflammatory lesions. Thus, none of the present 1000 patients was found with distant metastasis of thyroid cancer.

***Conclusions:*** Routine chest CT did not detect thyroid cancer lung metastasis in patients with PTMC. Thus, routine chest CT at the time of presentation is not required for patients with cT1aN0 PTCs.

## Introduction

Active surveillance has been recommended as an alternative management strategy to immediate surgery for patients with low-risk papillary thyroid microcarcinoma (PTMC) as established by the management guidelines of the Japan Association of Endocrine Surgery and former Japanese Society of Thyroid Surgery (1), as well as the 2015 American Thyroid Association guidelines (2). Low-risk PTMC patients include those without high-risk features such as clinical distant metastasis (M), lymph node metastasis (N), and significant extrathyroid extension (Ex) to the adjacent structures such as the trachea and recurrent laryngeal nerve. Implementation of these guidelines has generated favorable results as demonstrated not only from Japan (3–12) but also from the United States (13), Latin America (14,15), Korea (16,17), and Italy (18). In this context, evaluations conducted through imaging studies at the initiation of active surveillance are important for determination of high-risk features. The N and Ex features can be evaluated with ultrasound examination, and neck computed tomography (CT) may be added as necessary. If distant metastasis is present, the patient should be treated with total thyroidectomy followed by radioactive iodine (RAI) treatment. For accurately evaluating the M factor, systemic examinations with CT and positron emission computed tomography (PET-CT) may be necessary.

The most frequent site of distant metastasis of papillary thyroid cancer (PTC) is to the lung. However, it is unclear if chest CT is necessary at the initiation of active surveillance. At Kuma Hospital, we did not and do not routinely perform chest CT for candidates of active surveillance due to the presumed very low incidence of such events in these patients. In Western countries, the standard treatment modality for surgical cases of PTC is total thyroidectomy followed by RAI ablation, precluding the necessity of preoperative whole-body scans. Meanwhile, in Japan, less-than-total thyroidectomies have been frequently performed for patients with less advanced PTC, and RAI ablation is less frequently preformed. Accordingly, preoperative evaluation of the lung with chest CT has been an almost routine practice in most hospitals in Japan. At Kuma Hospital, we also routinely performed chest CT preoperatively in patients with PTC, including those with cT1aN0 disease.

The Japan Association of Endocrine Surgery and former Japanese Society of Thyroid Surgery recently performed a questionnaire survey on the current management of low-risk PTMC at their member institutions (19). Several respondents expressed concerns about whether and how a staging workup would be performed at the initiation of active surveillance. It remains an open clinical question as to whether a whole-body workup is needed for staging patients with cT1aN0 PTC who undergo nonsurgical active surveillance based on ultrasound evaluation. In fact, as described above, we have performed chest CT preoperatively in patients with PTC, including those with low-risk PTMC, but have not specifically investigated distant metastasis before active surveillance initiation. In this study, we aimed to evaluate the clinical value of preoperative chest CT for these patients through retrospective review of preoperative chest CT images in patients with cT1aN0 PTC.

## Materials and Methods

We evaluated consecutive patients who underwent surgery for PTMC, more specifically those with cT1aN0 PTC, between January 2006 and May 2012. All patients were diagnosed with PTC on preoperative cytology, and the diagnosis was confirmed on postoperative pathological examination. The indications for surgery included physician recommendation, patient preference, associated other benign diseases such as large thyroid nodules, Graves' disease, primary hyperparathyroidism, and tumor enlargement during previous active surveillance. Patients with high-risk features such as cervical lymph node metastasis or significant extrathyroid extension and those with coexisting thyroid malignancies other than PTC diagnosed cytologically or histopathologically were excluded from the present study. In total, 1000 patients (885 females and 115 males) with a median age of 55 years (range: 16–84 years) were included in this study. The median tumor size was 8 mm (range: 2–10 mm). All patients underwent preoperative chest CT (3- or 5-mm slice without contrast using Asteion™ Super 4 Edition or Activion™ 16; Toshiba Medical Systems Co., Tokyo, Japan). One author (S.K.) retrospectively reviewed all chest CTs and examined the study participant medical records. The Institutional Review Board at Kuma Hospital waived obtaining informed consent from the patients due to the retrospective nature of this study.

## Results

Of the 1000 consecutive patients, 326 (32.6%) were found to have various abnormal findings. The most frequent organ with abnormal findings was the lung (168 patients), followed by the liver (134 patients), breast (18 patients), gallbladder (15 patients), kidney (13 patients), cardiovascular lesion (7 patients), mediastinum (6 patients), spleen (4 patients), pleura (4 patients), pancreas (1 patient), and rib bone (1 patient). Thirty-five patients had abnormal findings in two organs, and six patients had abnormal findings in three organs.

**FIG. 1.**
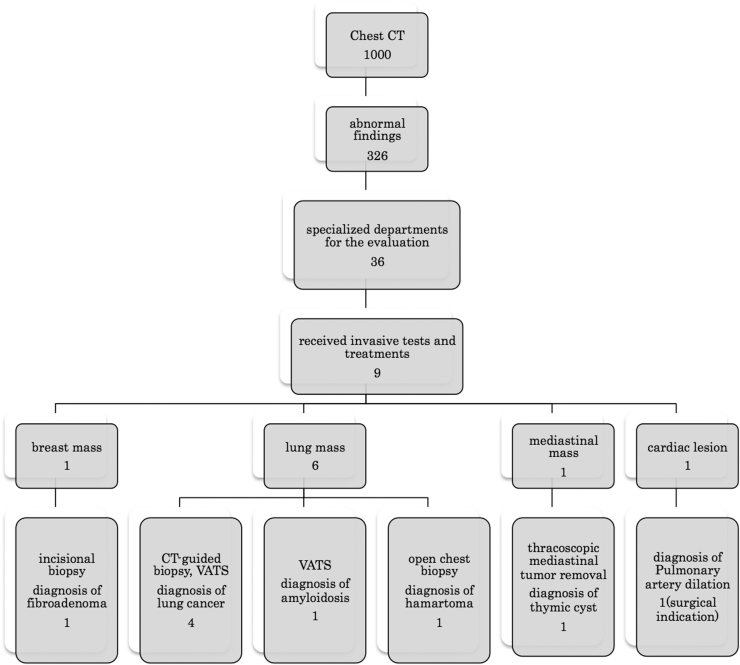
Patient management flowchart for abnormal findings on preoperative chest CT. None of the 1000 patients with cT1aN0 PTC was detected to have any distant metastasis of PTC, although 5 patients (0.5%, 4 with lung cancer and 1 with pulmonary artery dilation) benefited from the chest CT. The values indicate the number of patients. CT, computed tomography; PTC, papillary thyroid carcinoma.

Figure 1 shows the management flowchart for patients who had abnormal findings on chest CT. Thirty-six patients (3.6%) were referred to specialized departments for evaluation of abnormal lesions, and nine (0.9%) underwent invasive tests and/or treatments. One breast mass was diagnosed as fibroadenoma via incisional biopsy. Four of the six lung masses were diagnosed as lung carcinoma, and the remaining two were diagnosed as benign lesions via video-assisted thoracic surgery or open biopsy. One patient with a mediastinal mass underwent thoracoscopic mediastinal tumor removal and was diagnosed with a thymic cyst. One patient with cardiovascular lesion was recommended for surgery for pulmonary artery dilation (ventricular septal defect). In total, only five patients (0.5%; four patients with lung cancer and one with pulmonary artery dilation) benefited from the preoperative chest CT. More importantly, none of the 1000 patients had any distant PTC metastasis detected on preoperative chest CT.

The majority of the remaining 27 patients did not undergo further testing or interventions due to having presumed benign or inflammatory lesions. These included liver cysts in two patients, hepatic hemangioma in one patient, fibroadenoma of the breast in one patient, and mastopathy in two patients. Of the 21 patients with lung lesions, 8 patients had inflammatory changes, 1 had suspected hamartoma, 2 were suspected of having atypical mycobacteriosis, 9 were classified as having nonspecific findings, and 1 patient with suspected lung cancer was recommended for follow-up evaluations.

The remaining 290 patients who did not require any specific referrals had presumed benign or inflammatory lesions in the lung (141 patients), liver (131 patients), breast (14 patients), gallbladder (15 patients), kidney (13 patients), cardiovascular lesions (6 patients), mediastinum (5 patients), spleen (4 patients), pleura (4 patients), pancreas (1 patient), and rib bone (1 patient). Of these cases, eight with lung lesions, one with a liver lesion, and two with breast lesions had already undergone follow-up evaluations in other hospitals. Of the remaining cases, 37 with lung lesions and 1 with a breast lesion underwent observation in our hospital. Attending physicians evaluated these lesions and determined no requirement for further testing or follow-up evaluations. None of the present 1000 patients showed features suspicious for pulmonary metastasis such as increasing serum thyroglobulin or enlarging pulmonary nodules during follow-up.

## Discussion

In this study, of the 1000 patients with cT1aN0 PTC who underwent preoperative chest CT, abnormal findings in various organs were detected in 32.6%, but lesions requiring immediate treatments were found only in 0.5%. Furthermore, lung metastasis from PTC was not identified in any patient in this study.

Choi *et al.* have reported that none of the 4927 low-risk PTMC patients who underwent lobectomy with prophylactic central node dissection had distant metastases at surgery (20). Although they stated that patients were postoperatively followed up by ultrasound and serum thyroglobulin tests at 6- or 12-month intervals, and were made to undergo annual chest radiography or CT, the methods by which distant metastases were evaluated at the time of surgery are unclear. Reinke *et al.* did not detect distant metastasis in any of the 527 asymptomatic PTMC patients (21), but similar to the previous study, the methods used to preoperatively evaluate for distant metastases were also unclear.

The present study included 1000 consecutive patients with cT1aN0 PTC, which is characterized as low risk, and were evaluated for pulmonary metastasis using chest CT. Importantly, none of the 1000 patients had pulmonary metastasis, indicating that pulmonary metastasis of otherwise low-risk PTMC is an extremely rare event. It is well known that distant metastases to other organs are less frequent (22) and we can conclude that whole-body evaluation for distant metastasis using imaging studies at the initiation of active surveillance for low-risk PTMC is likely to not be necessary. In our study, the incidence of abnormal findings (32.6%) was high, but most of these findings were regarded as benign. Only 36 (3.6%) patients were referred to other departments for further follow-up studies, and 9 (0.9%) had invasive tests and/or treatments. However, five patients (0.5%; four with lung cancer and one with pulmonary artery dilation) presumably did benefit from the chest CT scan. This would suggest that the remaining 31 patients might have undergone unnecessary additional examinations, including invasive tests. These results also suggest that chest CT at the initiation of active surveillance for PTMC is not beneficial for patients with respect to cost, radiation exposure, and could subject individuals to unnecessary testing and invasive procedures resulting from incidental findings.

The Clinical Practice Guidelines for Breast Cancer by the Japanese Breast Cancer Society published in 2018 (23) reported that only 0.4% and 6.9% of patients with stage I and II breast cancer, respectively, were identified as having distant metastases via whole-body scans mainly through PET-CT. It was concluded that whole-body scans by CT and/or PET-CT are not recommended for stage I and II breast cancers since the incidence of distant metastasis in these patients is low. The results of the present study would suggest that the incidence of distant metastasis in patients with cT1aN0 PTC is even lower than in patients with stage I and II breast cancer.

This study has several limitations. Although our review of chest CTs from 1000 consecutive patients with low-risk PTMC did not identify any patient with pulmonary metastasis, the incidence of such events may be too low to be detected in such a cohort. One might correctly argue that chest CTs could be used to evaluate the overall pulmonary status at presentation in patients who develop pulmonary metastasis later during active surveillance. However, the number of cases that would receive such a benefit would be extremely rare and the overall small benefit when compared with the large number of drawbacks brings into question the utility of this approach.

In conclusion, distant metastases in patients with low-risk PTMC are extremely rare and were not detected on routine preoperative chest CT. Thus, our findings suggest that routine chest CT scanning at the time of presentation for evaluation of distant metastases is not required for patients with low-risk PTMCs.
